# The Continuum of Maternal Sepsis Severity: Incidence and Risk Factors in a Population-Based Cohort Study

**DOI:** 10.1371/journal.pone.0067175

**Published:** 2013-07-02

**Authors:** Colleen D. Acosta, Marian Knight, Henry C. Lee, Jennifer J. Kurinczuk, Jeffrey B. Gould, Audrey Lyndon

**Affiliations:** 1 National Perinatal Epidemiology Unit, University of Oxford, Oxford, United Kingdom; 2 Department of Pediatrics, Stanford University, Palo Alto, California, United States of America; 3 California Perinatal Quality Care Collaborative, Stanford, California, United States of America; 4 Department of Family Health Care Nursing, University of California San Francisco, San Francisco, California, United States of America; 5 California Maternal Quality Care Collaborative, Stanford, California, United States of America; UCL Institute of Child Health, University College London, United Kingdom

## Abstract

**Objective:**

To investigate the incidence and risk factors associated with uncomplicated maternal sepsis and progression to severe sepsis in a large population-based birth cohort.

**Methods:**

This retrospective cohort study used linked hospital discharge and vital statistics records data for 1,622,474 live births in California during 2005–2007. Demographic and clinical factors were adjusted using multivariable logistic regression with robust standard errors.

**Results:**

1598 mothers developed sepsis; incidence of all sepsis was 10 per 10,000 live births (95% CI = 9.4–10.3). Women had significantly increased adjusted odds (aOR) of developing sepsis if they were older (25–34 years: aOR = 1.29; ≥35 years: aOR = 1.41), had ≤high-school education (aOR = 1.63), public/no-insurance (aOR = 1.22) or a cesarean section (primary: aOR = 1.99; repeat: aOR = 1.25). 791 women progressed to severe sepsis; incidence of severe sepsis was 4.9 per 10,000 live births (95% CI = 4.5–5.2). Women had significantly increased adjusted odds of progressing to severe sepsis if they were Black (aOR = 2.09), Asian (aOR = 1.59), Hispanic (aOR = 1.42), had public/no-insurance (aOR = 1.52), delivered in hospitals with <1,000 births/year (aOR = 1.93), were primiparous (aOR = 2.03), had a multiple birth (aOR = 3.5), diabetes (aOR = 1.47), or chronic hypertension (aOR = 8.51). Preeclampsia and postpartum hemorrhage were also significantly associated with progression to severe sepsis (aOR = 3.72; aOR = 4.18). For every cumulative factor, risk of uncomplicated sepsis increased by 25% (95% CI = 17.4–32.3) and risk of progression to severe sepsis/septic shock increased by 57% (95% CI = 40.8–74.4).

**Conclusions:**

The rate of severe sepsis was approximately twice the 1991–2003 national estimate. Risk factors identified are relevant to obstetric practice given their cumulative risk effect and the apparent increase in severe sepsis incidence.

## Introduction

In countries with developed healthcare systems, sepsis remains a leading cause of preventable maternal morbidity and mortality [1]. Over the past decade the incidence of maternal deaths from severe maternal sepsis has increased in several European countries, most notably the United Kingdom[1]–[3]. There has also been an increase in the incidence and severity of sepsis morbidity in the general US and European populations[4]–[8]. While the absolute risk of maternal death from sepsis is small in the US (0.60 per 100,000 live births in the US; extrapolated from Berg et al [9]), the risk of severe sepsis morbidity is substantially larger (20.9 per 100,000 deliveries; extrapolated from Callaghan et al [10]).

Pregnant and peripartum women represent a particularly vulnerable population for developing sepsis because the maternal immune system is modulated during these periods [11]. The systemic inflammatory response syndrome (SIRS), an indicator of uncomplicated sepsis, is usually recognized by specific biomarkers [12]. However, physiological changes of pregnancy can mimic and sometimes mask these biomarkers and thus the pathophysiology of sepsis. Recognition of an infection can therefore be delayed until progression to severe sepsis [13]. For this reason, an understanding of the risk factors along the continuum of sepsis morbidity from uncomplicated sepsis to severe sepsis is important for targeting preventive strategies that could be implemented ‘upstream’ of severe sepsis. A population-based study examining the risk factors and outcomes of maternal sepsis morbidity has not been carried out in the US. Further, although maternal sepsis, severe sepsis and septic shock have been studied in isolation from one another in other countries, there has not been a study assessing how risk associated with these factors changes with the progression of severity.

The aim of this study was to investigate the incidence and risk factors of uncomplicated maternal sepsis, severe sepsis and septic shock as well as the probability of progression to severe sepsis, among all births from 2005 to 2007 in California, where approximately one in eight US births occur.

## Materials and Methods

### Data Source

This study was conducted using data linked from the California Vital Statistics records and statewide hospital discharge data from the Office of State Wide Planning and Development (OSHPD). This linked dataset contains comprehensive demographic and clinical information including mode of delivery and ICD-9-CM diagnosis and procedure codes from the birth hospitalization of essentially all inpatient live births in California. The dataset is described in detail in a previous study [14]. Data were linked using a probabilistic matching algorithm for large public health data sets and is described by Jaro [15], [16]. The linkage was performed by the California Perinatal Quality Care Collaborative under grant support from the March of Dimes. Linkage protocols and internal validation are described in a previous study [14]. Hospital identifiers were masked in order to anonymize the data.

### Ethics Statement

Institutional Review Board approval with waiver of consent for anonymous data was obtained from Stanford University and the University of California San Francisco.

### Study Design and Outcome Variables

This was a retrospective cohort study of maternal sepsis among all in-hospital live births in California between 2005 through 2007. Outcome variables were: uncomplicated sepsis, severe sepsis and septic shock. Women were grouped according to the most severe outcome. Cases of uncomplicated sepsis were identified as those with an ICD-9-CM code for septicemia (038.1–038.9) or sepsis (995.91). Cases of severe sepsis were those with an ICD-9-CM code for severe sepsis (995.92), or a sepsis code plus the management indicators: length-of-stay ≥90th percentile for mode of delivery (at least three days), or a postpartum transfer to intensive care [10], [14] or if the woman died. We adopted these management criteria for severe sepsis in order to account for potential misclassification of sepsis severity [10], since it was noted that a proportion of women with codes for septicemia or sepsis and no other severe morbidity had a long length-of-stay, other management indicators of severe morbidity, or were either transferred or died. These management criteria have been established in other morbidity studies [10], [14], as administrative datasets do not usually contain clinical information (such as heart rate, respiratory rate, white blood cell count, etc.), which would otherwise be used as clinical indicators of severe sepsis. We also regarded sepsis as severe if it was complicated by other acute co-morbidity and the woman had the criteria for severe sepsis (as defined above). Cases of septic shock were those with an ICD-9-CM code for septic shock (785.52). Women with multiple codes were categorized according to the most severe code; for example women with uncomplicated sepsis and severe sepsis codes were categorized as having severe sepsis, and women with uncomplicated or severe sepsis and septic shock codes were categorized as having septic shock. Final outcome groups were mutually exclusive.

Risk factors included demographic and clinical factors, other acute co-morbidities as well as management-based indicators of severe morbidity [17](Tables 1, 2, 3). When payor status was analyzed, public insurance (Medi-Cal; California Medicaid) was grouped together with no insurance in order to avoid under-reporting, because previous studies have found that up to 20% of women in California are uninsured during the first trimester of pregnancy before being later enrolled in a public program [18]. Adequacy of prenatal care was defined according to the Kotelchuck Adequacy of Prenatal Care Utilization Index; adequate prenatal care was defined as initiation of prenatal care within the first four months of pregnancy, and receipt of at least 80% of the expected number of prenatal care visits based on the American Congress of Obstetricians and Gynecologists (ACOG) prenatal care visitation standards for uncomplicated pregnancies [14], [19]. Other significant morbidities were identified according to ICD-9-CM diagnostic and procedure codes in addition to birth certificate diagnosis codes [14]. Chronic co-morbidities evaluated were diabetes and chronic hypertension. These conditions were identified according to ICD-9-CM codes for diabetes type I or II (diabetes mellitus) or gestational diabetes (ICD-9-CM codes 648.01–648.04 and 648.80–648.84), and chronic/pre-existing hypertension (essential hypertension, excluding preeclampsia/eclampsia) (ICD-9-CM codes 401–405). Maternal deaths were identified from OSHPD disposition data and Vital Statistics records. These data however did not include direct cause of death, and do not capture women who were readmitted with sepsis or complications of sepsis and subsequently died; therefore case fatality rates and risk factors for direct maternal death from sepsis could not be assessed in this study.

**Table 1:** Demographic characteristics of non-sepsis obstetric population compared to women with uncomplicated sepsis, compared to severe sepsis, compared to septic shock in California (2005–2007).

	Obstetric population without sepsis	P-value*	Uncomplicated sepsis	P-value*	Severe sepsis	P-value*	Septic shock
	n = 1620876		n = 807		n = 735		n = 56
**Maternal age**							
Median years (IQR)	28 (23–33)	0.001**	29 (24–33)	0.826**	29 (24–34)	0.423**	32 (22.5–35)
**Race**		0.055		0.017		0.26	
White	1265843 (78.1)		645 (79.9)		556 (75.7)		41 (73.2)
Black	86093 (5.3)		43 (5.3)		70 (9.5)		5 (8.9)
Asian	186749 (11.5)		70 (8.7)		66 (9.0)		9 (16.1)
Other/Multirace	82191 (5.1)		49 (6.1)		43 (5.9)		1 (1.8)
**Ethnicity**		0.032		0.049		0.768	
Non-Hispanic	750807 (47.0)		403 (50.8)		329 (45.7)		24 (43.6)
Hispanic	848365 (53.1)		391 (49.2)		391 (54.3)		31 (56.4)
**Education**		<0.0001		0.152		0.884	
High school or less	875186 (55.6)		491 (62.8)		427 (60.8)		29 (58.0)
Some college	556303 (35.4)		225 (28.8)		229 (32.6)		18 (36.0)
Some post-graduate	141612 (9.0)		66 (8.4)		46 (6.6)		3 (6.0)
**Health insurance**		0.761		0.005		0.313	
Private	767519 (47.5)		375 (46.5)		280 (38.4)		27 (48.2)
Military/Other government	49426 (3.1)		24 (3.0)		25 (3.4)		1 (1.8)
Public/uninsured	798983 (49.4)		407 (50.5)		425 (58.2)		28 (50.0)
**Hospital volume (deliveries per year)**		0.081		0.857		0.808	
<1000	116195 (7.2)		54 (6.7)		53 (7.2)		5 (8.9)
1000–3000	717022 (44.2)		332 (41.1)		303 (41.2)		21 (37.5)
≥3000	787659 (48.6)		421 (52.2)		379 (51.6)		30 (53.6)
**MSA population**		0.727		0.127		0.679	
Small (<250,000)	80856 (5.0)		44 (5.4)		28 (3.8)		3 (5.4)
Medium (250,000<1 million)	381495 (23.6)		183 (22.7)		146 (19.9)		13 (23.2)
Large (>1 million)	1157311 (71.5)		580 (71.9)		561 (76.3)		40 (71.4)

Figures are numbers (%) of women.

Categories are mutually exclusive.

MSA = Metropolitan Statistical Area.

* Difference in distribution between groups; χ^2^ test; Fisher's exact test for <5 observations.

** Wilcoxon rank-sum (Mann-Whitney) test.

**Table 2:** Clinical characteristics of non-sepsis obstetric population compared to women with uncomplicated sepsis, compared to severe sepsis, compared to septic shock in California (2005–2007).

	Obstetric population without sepsis	P-value*	Uncomplicated sepsis	P-value*	Severe sepsis	P-value*	Septic shock
	n = 1620876		n = 807		n = 735		n = 56
**Prenatal care**		0.13		0.593		0.292	
Adequate	1299448 (80.2)		630 (78.1)		582 (79.2)		41 (73.2)
Inadequate	321428 (19.8)		177 (21.9)		153 (20.8)		15 (26.8)
**Parity**		0.216		<0.0001		0.575	
Primiparous	625377 (38.6)		293 (36.5)		349 (47.6)		24 (43.6)
Multiparous	994185 (61.4)		510 (63.5)		385 (52.4)		31 (56.4)
**Multiple pregnancy**		0.626		<0.0001		0.66	
Singleton	1571423 (96.9)		780 (96.7)		657 (89.4)		49 (87.5)
Multiple	49453 (3.1)		27 (3.4)		78 (10.6)		7 (12.5)
**Chronic Co-Morbidities**							
Diabetes	113014 (7.0)	0.014	74 (9.2)	<0.0001	112 (15.2)	0.381	11 (19.6)
Chronic hypertension	15850 (1.0)	0.266	11 (1.4)	<0.0001	37 (5.0)	1.00	2 (3.6)
**Mode of delivery**		<0.0001		0.001		0.006	
Spontaneous vaginal	1037472 (64.0)		425 (52.7)		314 (42.7)		17 (30.4)
Primary caesarean	292114 (18.0)		232 (28.8)		272 (37.0)		34 (60.7)
Repeat caesarean	216915 (13.4)		120 (14.9)		123 (16.7)		4 (7.1)
Operative vaginal**	74375 (4.6)		30 (3.7)		26 (3.5)		1 (1.8)
**BMI (2007 only)**	*n = 481994*		*n = 250*		*n = 211*		n = 17
Median (IQR)	24.3 (21.5–28.3)	0.642***	24.5 (21.8–28.7)	0.298***	25.6 (22.1–29.3)	0.379***	24.2 (19.4–29.2)

Figures are numbers (%) of women.

* Difference in distribution between groups; χ2 test; Fisher's exact test for <5 observations.

** Forceps or vacuum extraction.

*** Wilcoxon rank-sum (Mann-Whitney) test.

**Table 3:** Other significant morbidity and maternal death among non-sepsis obstetric population compared to women with uncomplicated sepsis, compared to severe sepsis, compared to septic shock in California (2005–2007).

	Obstetric population without sepsis	P-value*	Uncomplicated sepsis	P-value*	Severe sepsis	P-value*	Septic shock
	n = 1620876		n = 807		n = 735		n = 56
**Postpartum LOS (median days; IQR)**							
Median days (IQR)	2 (2–3)	0.005**	2 (2–3)	<0.0001**	5 (4–10)	0.0003**	9.5 (5–18.5)
**Other significant morbidity**							
Preeclampsia	101575 (6.3)	0.252	57 (7.1)	<0.0001	155 (21.1)	0.491	14 (25.0)
Postpartum hemorrhage	45969 (2.8)	0.85	22 (2.7)	<0.0001	75 (10.2)	<0.0001	21 (37.5)
Wound complication	6031 (0.4)	0.562	2 (0.3)	<0.0001	68 (9.3)	0.219	2 (3.6)
*Cesarean section*	5093 (84.6)***		2 (100.0)***		65 (95.6)***		2 (100.0)***
3rd or 4th degree laceration	37915 (2.3)	0.977	19 (2.4)	0.313	12 (1.6)	1.00	1 (1.8)
Pelvic trauma	51080 (3.2)	0.931	25 (3.1)	0.24	31 (4.2)	0.72	1 (1.8)
Coagulation disorder	2087 (0.1)	1.00	1 (0.1)	<0.0001	40 (5.4)	<0.0001	16 (28.6)
Respiratory failure	1107 (0.1)	0.019	3 (0.4)	<0.0001	77 (10.5)	<0.0001	32 (57.1)
Renal failure	437 (0.03)	1.00	0 (0.0)	<0.0001	30 (4.1)	<0.0001	16 (28.6)
Heart failure	5007 (0.3)	1.00	2 (0.3)	0.001	18 (2.5)	<0.0001	11 (19.6)
**Management Indicators**							
Episiotomy	214552 (13.2)	<0.0001	66 (8.2)	0.119	45 (6.1)	0.329	2 (3.6)
Blood transfusion	11472 (0.7)	0.337	8 (1.0)	<0.0001	98 (13.3)	<0.0001	29 (51.8)
Hysterectomy	1344 (0.1)	0.488	1 (0.1)	<0.0001	14 (1.9)	<0.0001	6 (10.7)
Ventilation	762 (0.1)	0.009	3 (0.4)	<0.0001	56 (7.6)	<0.0001	29 (51.8)
**Maternal death**	108 (0.01)	1.00	0 (0.0)	0.012	6 (0.8)	<0.0001	8 (14.3)

Figures are numbers (%) of women.

* Difference in distribution between groups; χ2 test; Fisher's exact test for <5 observations.

** Wilcoxon rank-sum (Mann-Whitney) test.

*** Proportion of women who had a wound complication who had a cesarean section.

Incidence <0.1% in obstetric population and women with sepsis for: phlebitis or thrombophlebitis, pulmonary embolism, uterine rupture, anesthetic complications, dilation and curettage, and cerebrovascular disorders.

### Sample Size and Statistical Analyses

The sample size of this study was represented by the population incidence of maternal sepsis among virtually all hospitals in California; military hospitals and freestanding birth centers do not report discharge data (which comprised 1.9% (n = 31,884) of births). Data from these hospitals were therefore excluded, and the final analysis included the 1,622,474 births with reported discharge data. Frequencies of demographic and clinical variables and other significant morbidities were tabulated for uncomplicated sepsis, severe sepsis and septic shock case groups, and each group was compared to the immediately less severe group in the morbidity continuum [20]; uncomplicated sepsis was compared with all other women who gave birth in California without a sepsis code, severe sepsis was compared with uncomplicated sepsis and septic shock was compared with severe sepsis. Comparisons were made using chi square, Fisher’s exact and Wilcoxon rank-sum (Mann-Whitney) tests where appropriate.

There were no statistically significant differences in the proportion of *a priori* demographic and clinical factors (all factors except for mode of delivery) between severe sepsis and septic shock, and given that women with septic shock would have progressed through the severe sepsis stage, women with either of these outcomes were grouped together into a severe sepsis/septic shock outcome category. In order to evaluate the initial risk for developing sepsis, women with uncomplicated sepsis were compared with all other women who gave birth in California without a sepsis code. And in order to evaluate the risk for progression of sepsis severity, women with severe sepsis/septic shock were compared with women who had uncomplicated sepsis. All factors were initially compared using univariable logistic regression and then modeled using multivariable logistic regression. In the progression of severity model, with 783 cases of severe sepsis/septic shock compared to 815 cases of uncomplicated sepsis, and assuming a prevalence of exposure of at least 5.0% among women with uncomplicated sepsis, the analysis had 90% power at P<0.05 (two-sided) to detect a statistically significant odds ratio of 1.95 or greater.

Both multivariable models were constructed based on risk factors identified in previous literature and plausible confounding. All demographic and clinical factors were included in the models with the exception of metropolitan statistical area (MSA) and body mass index (BMI). Metropolitan statistical area was not included since it was found to be collinear with hospital volume. Body mass index was also not included since these data were only collected for one year, thus there was >60% missing data for this variable. The morbidities or management indicators: wound complication, coagulation disorder, organ system failures, blood transfusion, hysterectomy, and ventilation were not included because these factors were on the causal pathway of sepsis. Episiotomy, 3^rd^ or 4^th^ degree laceration and pelvic trauma were included in initial model iterations, but were removed because they were not significant in either univariable or multivariable regression, and did not affect the relationship between other predictor variables and the outcomes. In addition, both models were re-run with the ‘public/uninsured’ health insurance group disaggregated to test for any significant effects on the risk associated with other predictor variables (such as prenatal care) and the outcomes; no significant effects were detected. Likelihood ratio tests with a significance level of P<0.01 were used to check for interactions between demographic and clinical variables; no significant interactions were identified in the final adjusted model. The final models were adjusted for possible hospital clustering effect and were calculated using robust standard errors. Adjusted odds ratios (aOR) with 95% confidence intervals (CIs) are reported. Since all variables with the exception of those discussed above were included in the adjusted models, only results of the multivariable analysis are shown. Differences in rates reported in the text are all statistically significant. Stata SE statistical software 12.1 (StataCorp, College Station, TX) was used for all analyses.

## Results

During the study period from 2005 through 2007, there were 1,622,474 live births in California of which 1598 mothers developed sepsis. The absolute risk of developing sepsis was ten per 10,000 live births (95% CI = 9.4–10.3). Of all women who had sepsis, 807 had uncomplicated sepsis, 735 had severe sepsis, and 56 had septic shock. The absolute risk of all severe sepsis including septic shock was 4.9 per 10,000 live births (95% CI = 4.5–5.2). Of all inpatient maternal deaths, 14 mothers had severe sepsis/septic shock (11.5% of all maternal deaths over the study period).

The distributions of demographic and clinical characteristics among the non-sepsis obstetric population and the three sepsis outcome groups are shown in Tables 1–2 respectively. There were significant differences in age, education, ethnicity, presence of diabetes and mode of delivery between women with uncomplicated sepsis and the non-sepsis obstetric population. There were significant differences between women with severe sepsis compared to those with uncomplicated sepsis in race, ethnicity, health insurance status, parity, plurality, diabetes, hypertension and mode of delivery. However only mode of delivery differed between women with septic shock compared to severe sepsis, with a higher cesarean section rate for septic shock.

The distributions of other significant morbidities and maternal death among the non-sepsis obstetric population and the three sepsis outcome groups are shown in Table 3. The median length of postpartum hospital stay was progressively longer with increasing sepsis severity. The median length-of-stay for severely septic women without other acute co-morbidity was also five days (IQR = 4–10). Compared to women with uncomplicated sepsis, a larger proportion of women with severe sepsis had preeclampsia, postpartum hemorrhage and wound complications. The rate of preeclampsia was also more than three times higher among women with septic shock compared to women with severe sepsis (37.5% vs. 10.2% respectively; p<0.0001). The proportion of morbidities and management indicators of severe morbidity, which occur along the causal pathway of sepsis (wound complication, coagulation disorder, organ system failures, blood transfusion, hysterectomy, and ventilation) increased significantly with increasing sepsis severity. Nearly all wound complications across the spectrum of sepsis severity were among women who had a cesarean section. The case fatality of women with septic shock was 14.3% (95% CI = 6.4–26.2).

**Figure 1:**
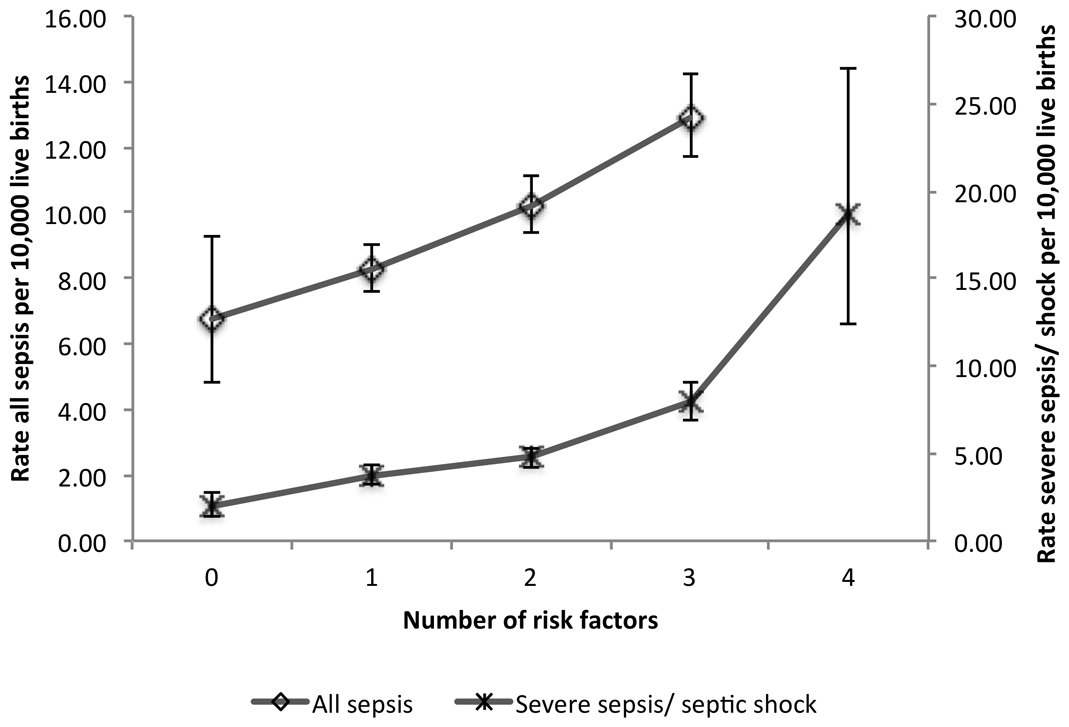
Absolute risk (95% CI) of all sepsis and severe sepsis/septic shock as a function of the number of *a priori* risk factors.

In assessing the risk of developing sepsis, compared to the non-septic obstetric population, women who were ≥25 years, who had high school or less education, or who had public or no health insurance were at significantly greater risk of developing uncomplicated sepsis, after adjustment for hospital clustering and all factors in the model (Table 4). Primary and repeat cesarean section were also associated with uncomplicated sepsis however the temporality of cesarean section vis-à-vis uncomplicated sepsis could not be determined. In assessing the risk of progression from uncomplicated sepsis to severe sepsis/septic shock, women who were of Black or Asian race, Hispanic ethnicity, who had public or no health insurance, diabetes, chronic hypertension, who delivered in low volume hospitals (<1,000 births per year), were primiparous or had a multiple pregnancy were at significantly increased risk of progression to severe sepsis. Risk associated with *a priori* demographic and clinical factors was significantly cumulative; compared to women without sepsis, for every additional factor, risk of uncomplicated sepsis increased by 25% (OR = 1.25; 95% CI = 1.17–1.32), and risk of progression to severe sepsis/septic shock increased by 57% (OR = 1.57; 95% CI = 1.41–1.74). The absolute risks for uncomplicated and severe sepsis in groups of women with multiple risk factors are shown in Figure 1.

**Table 4:** Adjusted odds ratios for factors associated with uncomplicated sepsis and severe sepsis/septic shock.

	Uncomplicated sepsis vs. no sepsis			Severe sepsis/shock vs. uncomplicated sepsis		
	aOR*	95% CI	P-value	aOR*	95% CI	P-value
**Maternal age**						
<18	0.94	(0.59–1.50)	0.805	1.2	(0.61–2.35)	0.594
18–24	1			1		
25–34	1.29	(1.08–1.54)	0.005	0.89	(0.68–1.18)	0.425
≥35	1.41	(1.12–1.78)	0.003	1.00	(0.71–1.40)	0.978
**Race**						
White	1			1		
Black	0.78	(0.55–1.09)	0.148	2.09	(1.34–2.26)	0.001
Asian	0.61	(0.47–0.79)	<0.0001	1.59	(1.07–2.37)	0.023
Other/Multirace	1.1	(0.77–1.52)	0.659	0.73	(0.43–1.23)	0.237
**Ethnicity**						
Hispanic	0.73	(0.61–0.88)	0.001	1.42	(1.09–1.83)	0.008
Non-Hispanic	1					
**Education level**						
High school or less	1.63	(1.35–1.97)	<0.001	0.79	(0.60–1.04)	0.089
More than high school	1			1		
**Health Insurance**						
Private	1			1		
Military/Other government	1.02	(0.67–1.56)	0.933	1.52	(0.85–2.72)	0.162
Public/uninsured	1.22	(1.02–1.46)	0.03	1.52	(1.19–1.94)	0.001
**Hospital volume (deliveries per year)**						
<1000	0.78	(0.58–1.04)	0.093	1.93	(1.15–3.23)	0.013
1000–3000	0.84	(0.72–0.98)	0.024	1.07	(0.85–1.35)	0.58
≥3000	1			1		
**Inadequate prenatal care**						
Yes	1.12	(0.94–1.33)	0.197	1.01	(0.78–1.30)	0.956
No	1					
**Primiparous**						
Yes	0.84	(0.71–1.00)	0.044	2.03	(1.56–2.63)	<0.0001
No	1			1		
**Multiple pregnancy**						
Yes	0.76	(0.51–1.12)	0.169	3.5	(2.09–5.85)	<0.0001
No	1			1		
**Diabetes**						
Yes	1.22	(0.95–1.56)	0.124	1.47	(1.04–2.09)	0.014
No	1					
**Chronic hypertension**						
Yes	1.23	(0.61–2.07)	0.491	8.51	(1.92–37.7)	0.005
No	1			1		
**Mode of Delivery**						
Spontaneous vaginal	1			1		
Primary caesarean	1.99	(1.68–2.34)	<0.0001	1.24	(0.97–1.59)	0.086
Repeat caesarean	1.25	(1.02–1.54)	0.035	1.33	(0.97–1.81)	0.076
Operative vaginal**	0.96	(0.66–1.41)	0.844	1.08	(0.62–1.90)	0.782
**Preeclampsia**						
Yes	0.99	(0.75–1.29)	0.921	3.72	(2.52–5.44)	<0.0001
No	1			1		
**Postpartum hemorrhage**						
Yes	1.00	(0.67–1.53)	0.967	4.18	(2.46–7.11)	<0.0001
No	1			1		

* Results adjusted for hospital clustering and for all factors listed in the table. Age, education level, hospital volume and parity treated as continuous linear terms in the analysis, but presented as categorical terms.

** Forceps or vacuum extraction.

## Discussion

This is the first population-based cohort study of the continuum of maternal sepsis severity in the US. We find that the rate of severe sepsis is approximately twice the estimated national rate, and that significant socioeconomic disparities exist among women who develop sepsis compared to those that do not. In addition to known clinical risk factors, public or no insurance, racial and ethnic minority status, low hospital birth volume, diabetes and chronic hypertension play a role in the risk of progression to severe sepsis/septic shock. Importantly, risk associated with a priori factors is significantly cumulative.

Callaghan and colleagues, in their study of severe morbidity during delivery hospitalizations in the US from 1991–2003, found that the rate of all severe morbidity was 5.1 per 1,000 deliveries, of which sepsis accounted for 4.1% [10]; this extrapolates to a severe sepsis rate of 2.1 per 10,000 deliveries. Using identical severity criteria, we found that the rate of all severe sepsis including septic shock in California from 2005 to 2007 was 4.9 per 10,000 live births. Factors that may partially contribute to this significantly higher rate are differing denominators (deliveries vs. live births), as well as inclusion of ICD-9-CM codes for sepsis, severe sepsis and septic shock, in addition to the ICD-9-CM code for septicemia. In our study, the additional sepsis codes accounted for 12.3% of severe cases. For comparison, even without these cases included, and taking into account the 0.6% [21] national rate of stillbirths (included in the ‘deliveries’ denominator), the severe sepsis rate would be approximately 100% higher than the previous national estimate. These results may therefore indicate a real increase in the incidence rate of severe sepsis in the obstetric population. Although we are unable to comment on whether there has been an increase in California specifically due to the lack of previous sepsis studies, there has been a significant increase in the rate of severe morbidity [14] and maternal death in California [22], indicating a likely increase in the rate of severe sepsis morbidity. Additionally, national rates of hospitalization for sepsis in the general population have more than doubled since 2000 [23].

Public or no insurance [24], racial and ethnic minority status[22], [25]–[27], and low hospital birth volume [14], [28] as risk factors for sepsis and progression to severe sepsis are consistent with other morbidity studies. Women with diabetes are at increased risk for maternal sepsis death [3], however the risk of severe sepsis morbidity has not been quantified at the population level. We found that diabetic women had 47% greater adjusted odds of progressing to severe sepsis compared to septic women without diabetes. This result represents the extension of previous findings that diabetic compared to non-diabetic women are at increased risk of infection during pregnancy and postpartum [29], [30].

The association between preeclampsia and certain types of infection, particularly urinary tract infection has been well established, however the mechanism of the association in still unclear [31]. Several studies suggest that infection plays a key role in initiation of preeclampsia or enhances the systemic inflammatory response [32], [33]. Although it is unclear whether infection is causative of preeclampsia, results of this study and another recent study from the UK [12], indicate a strong association between the inflammatory process of sepsis and preeclampsia.

Interestingly, we also found that chronic (preexisting) hypertension, independent of preeclampsia, was a strong risk factor for progression to severe sepsis. Chronic hypertension has not been demonstrated to be a risk factor for maternal sepsis in previous studies. This result may be attributed to the significant increase in chronic hypertension in the obstetric population [34], however the population prevalence was still very low. Although sparsely described for the obstetric population, the pathogenesis of severe sepsis and septic shock can differ between normotensive and hypertensive women. A sign of severe sepsis is hypoperfusion which is marked by hypotension, however women with chronic hypertension may develop critical hypoperfusion at a higher blood pressure, and therefore earlier, than normotensive women [35].

Established risk factors for developing sepsis supported by this study include older maternal age and cesarean section [2], [36]. Given that prophylactic antibiotics should have been in general practice during the study period, it is possible that a proportion of women undergoing a cesarean section may have had an infection prior to delivery, although there was also a strong association between cesarean section and wound complication indicating postoperative infection as well. Changes in recommendations for the timing of prophylactic antibiotics administration to pre-incision, as apposed to intraoperative were implemented in 2010 by the ACOG; it is the standard practice in non-obstetric surgery [37] and clinicians should adhere to this guideline as it has been demonstrated to reduce the rate of postoperative infection [37]. Primiparity, multiple births and postpartum hemorrhage were all associated with progression to severe sepsis and have also been identified as risk factors for severe sepsis in previous studies [2], [38].

Our results should be considered in light of several limitations. First, data used in this study are subject to possible inaccuracies inherent in administrative datasets. Although it was impossible to audit potential misclassification, it is likely that the large sample size mitigates random errors, while adjustment for hospital clustering accounts for systematic reporting errors at the hospital level. As body mass index was only available for one year, it was not possible to adjust for the potential confounding effect of obesity [12], particularly with regards to diabetes and hypertension. Additionally, it was not possible to assess the temporality of factors such as mode of delivery and acute comorbidities with respect to sepsis, or to comment on the effect of labor induction on comorbidities with respect to sepsis because we did not have access to this variable. There was also insufficient power to exclude the role of chance in the lack of association between maternal age ≥35 with the odds of progression to severe sepsis. Lastly, elements such as Hispanic ethnicity may not be representative of the wider US population.

Results of the study have significant implications for heath policy and obstetric patient care, particularly in light of an apparent increase in incidence and the significantly cumulative effect that *a priori* factors have on the risk of progression along the sepsis severity continuum. Clinical risk factors such as primiparity, multiple birth, diabetes and cesarean section have been incorporated into obstetric guidelines for sepsis in other countries [39], [40], however, there are currently no national obstetric clinical guidelines for prevention and management of obstetric sepsis in the US. Risk of deterioration associated with these factors, which may complicate management, must also be considered. Chronic hypertension with possible early hypoperfusion, and high risk for preeclampsia must also be considered in obstetric sepsis guidelines. Lastly, socioeconomic and racial disparities associated with the risk of progression to severe sepsis clearly exist and must be addressed at public health policy and patient care levels.
